# Perioperative Systemic Therapy in Rare, Chemosensitive Subtypes of Retroperitoneal Sarcoma: A Hospital-Based Propensity Score-Matched Analysis

**DOI:** 10.3390/cancers17121931

**Published:** 2025-06-10

**Authors:** Benjamin Wiesler, Laleh Forountani, Amir Ashraf Ganjouei, Lara Studerus, Christoph Kettelhack, Fatime Krasniqi, Benjamin Kasenda, Beat P. Müller, Mohamed A. Adam, Alexander Wilhelm

**Affiliations:** 1 Department of Surgery, Clarunis–St. Clara Hospital & University Hospital Basel, 4031 Basel, Switzerland; benjamin.wiesler@clarunis.ch (B.W.); lara.studerus@clarunis.ch (L.S.);; 2 Surgical Outcome Research Center Basel, University Hospital Basel, University Basel, 4031 Basel, Switzerland; 3 Department of Surgery, University of California San Francisco, San Francisco, CA 94143, USA; laleh.foroutani@ucsf.edu (L.F.); amir.ashrafganjouei@ucsf.edu (A.A.G.); 4 Department of Medical Oncology, University Hospital Basel, 4031 Basel, Switzerland

**Keywords:** retroperitoneal sarcoma, chemotherapy, overall survival, propensity score matching, NCDB

## Abstract

**Background:** There is an ongoing debate about the role of perioperative chemotherapy in retroperitoneal sarcoma (RPS). The aim of this study was to evaluate the effectiveness of perioperative chemotherapy in subtypes of RPS that are considered chemosensitive, including retroperitoneal angiosarcoma, undifferentiated pleomorphic sarcoma, myxoid liposarcoma, spindle cell sarcoma, and synovial sarcoma. **Methods:** This is a population-based retrospective cohort study. Patients with localized retroperitoneal sarcoma who underwent surgery were included from the US National Cancer Database (NCDB). After propensity score matching for the factors age, sex, grade, margin status, and tumor size, multivariable logistic and Cox regression analyses were used to identify factors associated with systemic therapy and their potential impact on the survival of patients with localized RPS. **Results:** We included 851 patients who underwent surgery between 2004 and 2020 (85% white, 41% female, and mean age 62 years). Of those, 227 patients (26.7%) received perioperative chemotherapy. In multivariable logistic regression, age ≤ 60 and tumor grading GIII/IV vs. GI/II were associated with a higher probability of receiving perioperative chemotherapy. After propensity score matching, we detected no difference in overall survival between patients who received chemotherapy and those who did not (HR 0.89, CI 0.55–1.43; and log-rank *p* = 0.92). Patient age ≤80 and tumor grading GI/II vs. GIII/IV were associated with improved overall survival. **Conclusions:** In this large analysis, the use of perioperative chemotherapy was not associated with improved survival in rare, chemosensitive subtypes of retroperitoneal sarcoma. However, selection bias must be considered when interpreting these findings.

## Simple Summary

The benefits of perioperative chemotherapy for retroperitoneal sarcoma are the subject of ongoing research. The aim of this retrospective, hospital-based analysis was to evaluate the possible benefit of the use of perioperative chemotherapy in rare subtypes of retroperitoneal sarcoma that are considered chemosensitive. A total of 851 patients were included who underwent surgery between 2004 and 2020 using the National Cancer Database of the United States. No clear benefit of perioperative chemotherapy was observed in this analysis. However, this study has a number of limitations that limit the generalizability of these findings.

## 1. Introduction

Retroperitoneal sarcoma (RPS) is a rare disease, representing 1% of all solid malignancies [1,2]. RPS exhibits substantial histologic heterogeneity, with liposarcoma, leiomyosarcoma, and undifferentiated sarcoma representing the most prevalent entities [2]. En-bloc resection is regarded as the gold standard of treatment. Macroscopic total excision is feasible in approximately 50–70% of patients [3]. However, recurrence rates remain high, with up to 50% local recurrences within five years and 20–50% distant recurrences, depending on histologic subtypes and other prognostic characteristics [3].

The use of neoadjuvant and adjuvant therapies remains a subject of ongoing discourse. While the use of neoadjuvant radiotherapy was recently assessed in a large prospective randomized trial (STRASS trial), there is a lack of prospective randomized trials evaluating the effect of perioperative chemotherapy in RPS [4]. Several retrospective population-based studies have not demonstrated a clear benefit of perioperative chemotherapy, be it neoadjuvant or adjuvant [5,6,7,8]. One prospective randomized trial showed a potential benefit of regional hyperthermia in combination with chemotherapy for local control and disease-specific survival in macroscopically resected retroperitoneal and abdominal sarcoma [9]. Nevertheless, a considerable number of these studies are subject to high selection bias. Consequently, the Australian National Health and Medical Research Council (NHMRC) hierarchy assesses the current evidence level as Level III-2 [10]. Due to this lack of evidence, the most recent guidelines from the European Society for Medical Oncology (ESMO) suggest that the administration of chemotherapy should only be considered in selected cases, such as borderline resectable RPS and RPS considered sensitive to chemotherapy, e.g., synovial sarcoma [11].

The STRASS 2 trial is the first prospective, randomized trial to evaluate the effect of neoadjuvant chemotherapy in patients with retroperitoneal sarcoma [12]. It has been recruiting since 2023. However, the STRASS 2 trial exclusively includes patients diagnosed with dedifferentiated liposarcoma and leiomyosarcoma. However, other sarcoma subtypes have been demonstrated to be susceptible to chemotherapy, including synovial sarcoma [11]. But because of their rarity, inclusion into randomized trials remains a challenge. Consequently, the necessity arises for large, population-based studies to assess the effectiveness of perioperative chemotherapy in these specific sarcoma subtypes. The objective of the present population-based study was to evaluate the impact of perioperative chemotherapy in rare types of retroperitoneal sarcoma using the National Cancer Database (NCDB).

## 2. Methods

### 2.1. Data Source and Study Population

This retrospective cohort study utilized data from the National Cancer Database (NCDB) from 2004 to 2021. The NCDB, a hospital-based oncology database jointly sponsored by the American College of Surgeons and the American Cancer Society, captures approximately 70% of all newly diagnosed cancer cases in the United States [13]. We included adult patients (≥18 years old) diagnosed with retroperitoneal sarcoma (RPS) who underwent surgical resection and had one of the following histological subtypes identified using ICD-O-3 codes: synovial sarcoma (9040/3, 9041/3, 9042/3, 9043/3), spindle cell sarcoma (8801/3), undifferentiated pleomorphic sarcoma (8802/3), myxoid/round cell liposarcoma (8852), and angiosarcoma (9120). Patients with incomplete records regarding systemic chemotherapy receipt and survival outcomes were excluded. Due to the low number of cases, patients who underwent neoadjuvant and adjuvant chemotherapy were included. In the NCDB, adjuvant and neoadjuvant therapies are classified as perioperative if they were administered within 180 days of surgery. The NCDB does not include information on the chemotherapy regimens administered. Consequently, these variables were excluded from the analysis.

### 2.2. Statistical Analysis

Descriptive statistics summarized patient and tumor characteristics using medians with interquartile ranges (IQRs) for continuous variables and proportions for categorical variables. Differences between chemotherapy and non-chemotherapy groups were assessed using Student’s *t*-tests for continuous variables and chi-square tests for categorical variables. To adjust for confounding, propensity score matching (PSM) was performed using nearest-neighbor matching without replacement, applying a caliper width of 0.2 [14]. Matching covariates included age, sex, tumor size, tumor grade, and margin status. For propensity score matching, the “MatchIt” package in R Version 4.4.1 (R) was used.

Kaplan–Meier survival curves estimated overall survival, and the log-rank tests compared groups in unmatched and matched cohorts. Additional stratified analyses evaluated survival by tumor grade and histologic subtype. Multivariable Cox regression models were constructed to evaluate the association of variables such as age, tumor size, tumor grade, chemotherapy, margin status, and stage with survival in the overall cohort and in stratified subgroup analysis for each subtype of RPS separately. Age and tumor size was evaluated as categorical variables. Multivariable logistic regression was used to assess predictors of chemotherapy receipt, incorporating variables such as age, sex, tumor size, tumor grade, and radiotherapy use. The results were reported as odds ratios (ORs) with 95% confidence intervals (CIs). All statistical analyses were conducted using R version 4.4.1 (R), and a *p*-value < 0.05 was considered statistically significant.

## 3. Results

### 3.1. Demographic, Clinical, and Pathologic Characteristics

**Figure 1:**
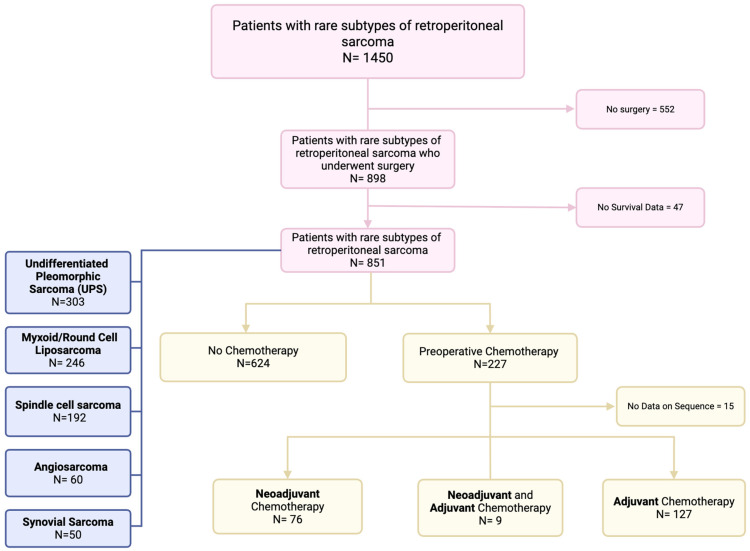
Participant flow diagram.

A total of 851 patients were included in the study, of whom 227 (27%) received perioperative systemic chemotherapy, while 624 (73%) underwent surgery without chemotherapy (Figure 1). The median age of the overall cohort was 62 years (IQR: 51–71). Patients who received chemotherapy were significantly younger than those who did not (56 years [IQR: 42–64] vs. 64 years [IQR: 55–73], *p* < 0.05). The sex distribution was similar between the groups, with 41% of patients being female and 59% male (*p* = 0.9) (Table 1).

The distribution of histologic subtypes differed significantly between patients who received chemotherapy and those who did not (*p* < 0.05) (Table 1). The rate of patients with synovial sarcoma who received chemotherapy was 78%. Conversely, the proportion of patients with myxoid/round cell liposarcoma and undifferentiated pleomorphic sarcoma who received chemotherapy was only 12% and 29%, respectively. Tumor characteristics also varied between groups (Table 1). The rate of high-grade tumors (Grade III/IV) was higher in the chemotherapy group (61% vs. 46%). Among chemotherapy recipients, 33% of patients received neoadjuvant chemotherapy, 56% received adjuvant chemotherapy, and 4% received both neoadjuvant and adjuvant therapy. Final surgical margin status did not show a statistically significant difference between the two groups.

### 3.2. Multivariable Analysis

Multivariable logistic regression analysis demonstrated that age and tumor grade were independent predictors of treatment with chemotherapy (Table 2). Compared to younger patients (<60 years), older patients were less likely to receive chemotherapy (age 60–79: OR 0.56 [95% CI: 0.32–0.97], *p* < 0.05; age ≥ 80: OR 0.18 [95% CI: 0.04–0.82], and *p* < 0.05). In contrast, patients with high-grade tumors (Grade III/IV) were more likely to receive chemotherapy (OR 2.52 [95% CI: 1.35–4.69], *p* < 0.05).

### 3.3. Survival Analysis

**Figure 2:**
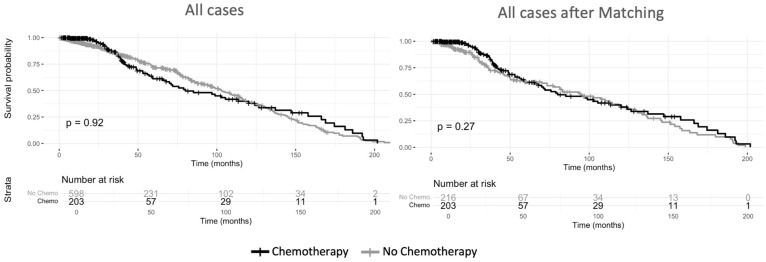
Overall survival of patients with localized retroperitoneal sarcoma. All cases before and after propensity score matching.

**Figure 3:**
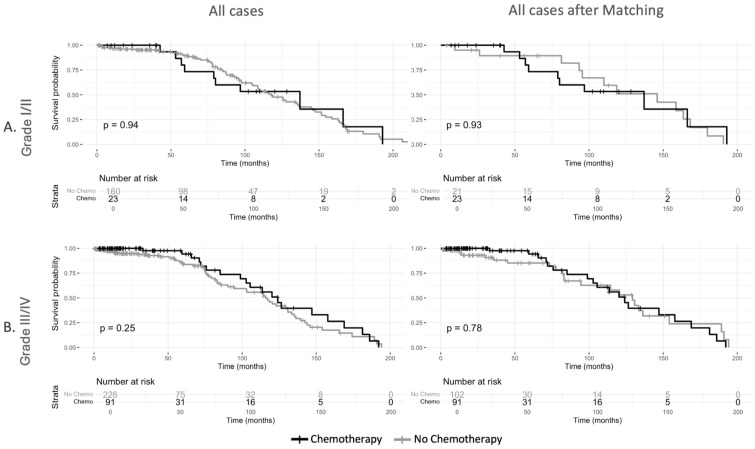
Overall survival of patients with localized retroperitoneal sarcoma (**A**). Subgroup analysis of patients with Grade I/II retroperitoneal sarcoma before and after propensity score matching (**B**). Subgroup analysis of patients with Grade III/IV sarcoma before and after propensity score matching.

Kaplan–Meier survival analysis in the unmatched cohort demonstrated no difference in overall survival between patients who received chemotherapy and those who did not (log-rank *p* = 0.92) (Figure 2). When stratified by tumor grade, no significant survival differences were observed between the chemotherapy and non-chemotherapy group for patients with Grade I/II tumors (log-rank *p* = 0.94) or Grade III/IV tumors (log-rank *p* = 0.25) (Figure 3).

**Figure 4:**
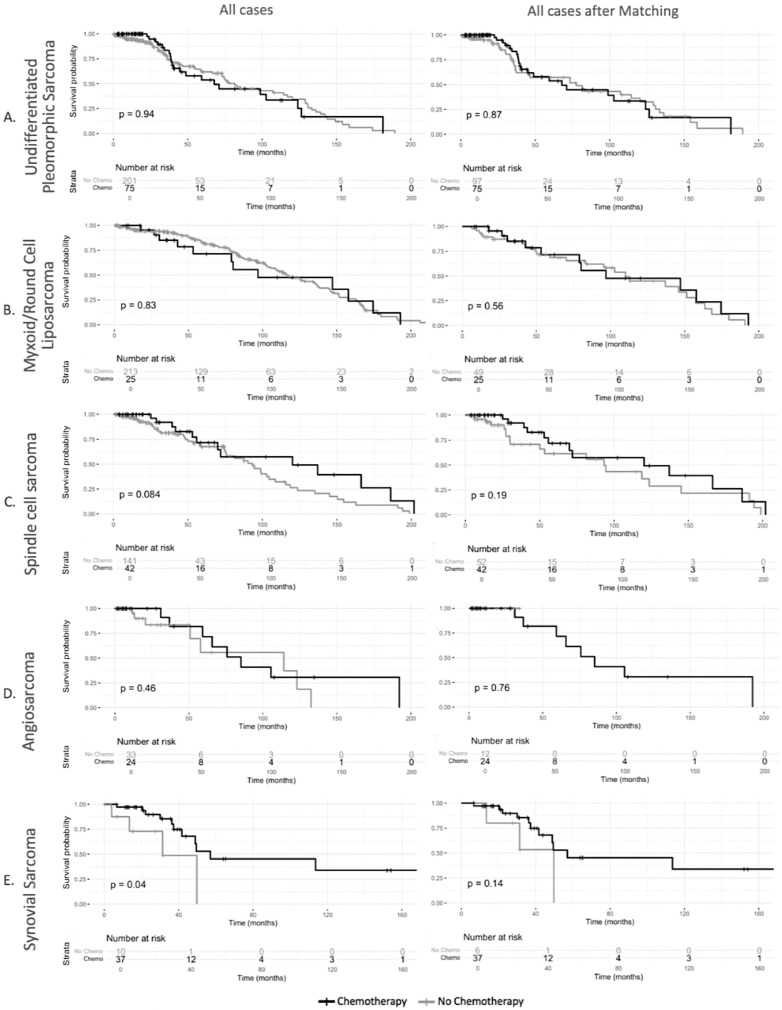
Overall survival of patients with localized retroperitoneal sarcoma in different subgroups of retroperitoneal sarcoma (**A**). Patients with undifferentiated pleomorphic sarcoma before and after propensity score matching (**B**). Patients with myxoid cell sarcoma before and after propensity score matching (**C**). Patients with spindle cell sarcoma before and after propensity score matching (**D**). Patients with angiosarcoma before and after propensity score matching (**E**). Patients with synovial sarcoma before and after propensity score matching.

Following propensity score matching, survival analyses remained consistent with the unadjusted findings, demonstrating no difference in overall survival between chemotherapy recipients and non-recipients (log-rank *p* = 0.27) (Figure 2). Similarly, tumor grade-stratified survival analyses in the matched cohort showed no differences for Grade I/II tumors (log-rank *p* = 0.93) or Grade III/IV tumors (log-rank *p* = 0.78) (Figure 3). There were no differences in survival outcomes between patients who received neoadjuvant chemotherapy and those who received adjuvant chemotherapy (Supplementary Figure S1). Supplementary Figure S2 shows the sample balance before and after propensity score matching for age, sex, grade, margin status, and tumor size. The effect of chemotherapy on survival varied across histologic subtypes (Figure 4). In the unmatched cohort, Kaplan–Meier survival analysis demonstrated no overall survival benefit for chemotherapy in undifferentiated pleomorphic sarcoma (log-rank *p* = 0.94), myxoid/round cell liposarcoma (log-rank *p* = 0.83), and angiosarcoma (log-rank *p* = 0.46). In spindle cell sarcoma, treatment with chemotherapy showed a trend toward improved survival, though the difference was not statistically significant (log-rank *p* = 0.08). However, in patients with synovial sarcoma, perioperative chemotherapy was associated with an improvement in survival (log-rank *p* = 0.04).

Following propensity score matching, survival outcomes remained broadly consistent with the unadjusted analysis (Figure 4). Chemotherapy was not associated with improved survival in undifferentiated pleomorphic sarcoma (log-rank *p* = 0.87), myxoid/round cell liposarcoma (log-rank *p* = 0.56), spindle cell sarcoma (log-rank *p* = 0.19), and angiosarcoma after matching (log-rank *p* = 0.76). Similarly, in patients with synovial sarcoma where the unadjusted analysis suggested an overall survival benefit for perioperative chemotherapy, Kaplan–Meier survival analysis did not show an association of chemotherapy with overall survival (log-rank *p* = 0.14).

Cox proportional hazards regression analysis of the entire cohort did not identify an association of survival with perioperative chemotherapy (HR 0.89 [95% CI: 0.55–1.43], *p* = 0.63) (Table 3). Older age (≥80 years) compared to age < 60 was associated with reduced survival (HR 5.05 [95% CI: 1.85–13.79], *p* < 0.05), as was a macroscopic residual tumor (R2 margin) following surgery compared to R0 margin status (HR 2.77 [95% CI: 1.18–6.53], *p* < 0.05). Tumor size, grade, sex, and the receipt of radiotherapy were not associated with survival.

Subgroup analyses revealed histology-specific effects. In undifferentiated pleomorphic sarcoma, chemotherapy was not associated with survival (HR 1.43 [95% CI: 0.58–3.50], *p* = 0.44), while positive surgical margins were predictors of worse survival outcomes (Table S1). Similarly, in myxoid liposarcoma, chemotherapy was not associated with a survival advantage (HR 1.04 [95% CI: 0.43–2.49], *p* = 0.93) (Table S2). Additional subgroup analyses for spindle cell sarcoma, angiosarcoma, and synovial sarcoma did not identify significant or meaningful correlations for survival due to the small sample sizes in these groups.

## 4. Discussion

This large, propensity score-matched analysis is the first study to evaluate the potential impact of perioperative chemotherapy in rare subtypes of retroperitoneal sarcoma that are considered sensitive to chemotherapy. The administration of perioperative chemotherapy did not demonstrate a survival benefit within the overall cohort or in the subgroup analysis of the various entities. The unmatched analysis of patients with synovial sarcoma suggested an improvement in overall survival. However, this survival benefit was no longer observed after propensity score matching. In multivariable Cox proportional hazard regression, older age and R2 resection were found to be associated with reduced overall survival. In multivariable logistic regression, younger age (age < 60 years) and higher grade (Grade III/IV) were associated with the use of perioperative chemotherapy.

The impact of perioperative chemotherapy on the overall survival of patients with RPS has been examined in several retrospective studies. A systemic review of 22 retrospective studies and a meta-analysis showed that most of the studies lacked detailed information on histology, treatment regimens, and provided baseline characteristics [10]. The collective findings of these studies indicated that perioperative chemotherapy did not demonstrate a significant association with enhanced outcomes, specifically in terms of local recurrence, metastasis-free survival, disease-free survival, or overall survival, in patients with primary, localized, and resectable RPS [10]. The most recent NCDB-based analysis, conducted by Tortorello et al., exclusively included patients diagnosed with high-grade leiomyosarcoma (LMS) and dedifferentiated liposarcoma. This study encompassed a total of 2656 patients [15]. The analysis revealed no beneficial effect of neoadjuvant chemotherapy in these subtypes of RPS [15]. Due to an absence of randomized trials, the current NCCN and ESMO Guidelines are based on the findings of soft tissue sarcoma of the extremity [11,16]. Nonetheless, the findings of these studies are incongruent. One older randomized study that compared the effectiveness of surgery alone with that of neoadjuvant chemotherapy followed by surgery in 134 patients with high-risk soft tissue sarcoma did not demonstrate a significant survival benefit in the group that received neoadjuvant chemotherapy [17]. In contrast, another randomized trial among patients with high-grade or recurrent extremity sarcoma demonstrated a median disease-free survival and overall survival benefit in the group of patients that received adjuvant chemotherapy with epirubicin and ifosfamide compared to surgery alone with an absolute overall survival benefit after adjuvant chemotherapy of 19% at four years [18]. However, the findings derived from soft tissue sarcoma of the extremities and trunk may not be directly applicable to retroperitoneal sarcoma. The ongoing STRASS II trial is the first randomized trial to evaluate the use of neoadjuvant chemotherapy in patients with high-risk RPS. The trial will include 250 patients diagnosed with leiomyosarcoma or high-grade liposarcoma, who will be randomly assigned to receive either neoadjuvant chemotherapy and surgery or surgery alone. Patients diagnosed with leiomyosarcoma will receive a combination of Doxorubicin and Dacarbazine, while those with high-grade liposarcoma will receive a regimen of Doxorubicin and Ifosfamide. In the present analysis, a subset of rare entities that are not suitable for inclusion in randomized trials due to their rarity was evaluated using a population-based approach, which represents the only feasible method for evaluating these entities. In the present analysis, no correlation between perioperative chemotherapy and overall survival could be demonstrated for these entities, apart from a beneficial effect in the unmatched analysis for synovial sarcoma.

The current ESMO guidelines recommend treatment with perioperative chemotherapy in patients diagnosed with synovial sarcoma, as these tumors are considered to be chemosensitive [11]. In accordance with these recommendations, the proportion of patients who received perioperative chemotherapy was found to be significantly higher in patients with synovial sarcoma than in patients with other subtypes of RPS in the current analysis. The assumption that synovial sarcoma are more chemosensitive than other soft tissue sarcomas is, among others, based on pooled data from 15 studies of advanced soft tissue sarcomas [19]. Nevertheless, the debate regarding which subgroups of patients with synovial sarcoma would most benefit from systemic therapy remains active [20,21]. The treatment with perioperative chemotherapy was associated with a survival benefit among patients diagnosed with synovial sarcoma in the unmatched analysis of the current study. However, after propensity score matching, this observed effect was no longer evident. This result is limited by the reduced sample size after matching and further suggests the possibility of confounding factors influencing the initial findings in the unmatched cohort. After propensity score matching, only six patients with synovial sarcoma who did not undergo chemotherapy remained for comparison. Furthermore, including or excluding ifosfamide from the perioperative chemotherapy regimen may affect the results, particularly given that ifosfamide has been shown to be more effective in treating synovial sarcoma than other histological types [20]. Since the NCDB does not contain information on specific drugs and chemotherapy regimes, it remains unclear to what extent this influences the results.

Treatment with perioperative chemotherapy was more likely in younger patients and patients with higher grades in the current analysis. The impact of age and grading on survival outcomes in patients with retroperitoneal sarcoma has been well documented [22]. For instance, a retrospective cohort study conducted by Avances et al. demonstrated that higher histological grading is associated with an elevated local recurrence rate. Local recurrence has been identified as a critical factor in the determination of mortality in patients diagnosed with retroperitoneal sarcoma [23]. This may serve as one possible rationale for the administration of chemotherapy to patients diagnosed with high-grade RPS. There are several nomograms available that predict outcomes for patients with RPS, all of which incorporate age and grade in their predictive models for overall and disease-specific survival [24,25,26]. Among these, the nomogram developed by Gronchi et al. has undergone external validation and is recommended by the American Joint Committee on Cancer (AJCC) as a staging system [26]. There is a need to incorporate innovative markers and methods into nomograms to improve their prognostic ability [27]. Due to a lack of detailed data, it was not possible to examine whether patients who received chemotherapy would have benefited from chemotherapy according to the recommendations from the “SARCULATOR” [27,28].

It is reasonable to hypothesize that not all histological subtypes respond in a uniform manner to standard chemotherapy regimens [29]. A multicenter randomized trial was conducted to evaluate the efficacy of a histotype-tailored chemotherapy regimen, which consisted of the following drugs: trabectedin for myxoid liposarcoma, gemcitabine plus dacarbazine for leiomyosarcoma, ifosfamide plus etoposide for malignant peripheral nerve sheath tumor, high-dose ifosfamide for synovial sarcoma, and gemcitabine and docetaxel for undifferentiated pleomorphic sarcoma. No difference was observed in disease and overall survival after 12 months when compared to standard chemotherapy [30]. Nevertheless, the trial had to be closed prematurely. In the present analysis, it was not possible to include the histological subtype in the Cox proportional hazard regression model as a covariable, as it was not clear which subtype should serve as a reference. It is reasonable to assume that the histological subtype is a determining factor in the response to the standard chemotherapeutic regimen. Moreover, the proportion of patients who received chemotherapy differed across the various subtypes. Synovial sarcoma was identified as the most prevalent subtype, with a significant proportion of patients receiving chemotherapy. It is possible that this introduced additional bias into the analysis and had a direct impact on the overall results.

There are a number of other limitations to the study: It must be acknowledged that the study’s retrospective design inherently introduces several limitations, including the possibility of miscoding. Even with matching, a retrospective study is still prone to bias. Furthermore, the use of a national registry necessitates the acceptance of missing variables, which can compromise the precision of the data. However, we excluded all patients with incomplete records regarding systemic chemotherapy receipt and survival outcomes. Additionally, the retrospective analysis is constrained to the description of associations rather than the establishment of causal relationships. The NCDB lacks information on the exact chemotherapy regimens used. In addition, there is a lack of detail on the extent of surgery, the duration of chemotherapy, and whether the chemotherapy could be completed. Furthermore, the NCDB lacks data on recurrence rates, the site of recurrence, and disease-specific survival rates. Only patients treated in cancer-accredited hospitals are included in the NCDB. Some patients treated in non-accredited hospitals may be missed. This could have resulted in selection bias. Additionally, despite using one of the largest databases available, only a small number of patients could be enrolled. Considering that the NCDB dataset represents one of the largest datasets of RPS in the world, it seems very difficult to investigate a larger cohort. Given the limited number of cases in the various cohorts, it was not feasible to adjust for the distinct histological subtypes in the Cox and logistic regression models. This may have introduced an additional selection bias. This is a particularly salient consideration when evaluating the varying rates of patients who have undergone chemotherapy across the various histologic subtypes.

The use of perioperative chemotherapy in RPS is a highly relevant but challenging topic. Currently available evidence is limited, and perioperative chemotherapy in RPS is part of ongoing research. This is the first study to evaluate the effect of perioperative chemotherapy in rare retroperitoneal sarcoma subtypes that are considered sensitive to chemotherapy, including angiosarcoma, undifferentiated pleomorphic sarcoma, myxoid liposarcoma, spindle cell sarcoma, and synovial sarcoma. Although perioperative chemotherapy is widely used in this setting, particularly for synovial sarcoma, the routine use of perioperative chemotherapy in the entities studied is not supported by our data and may be questioned. The results regarding the use of perioperative chemotherapy in synovial sarcoma are inconclusive, most likely due to the rarity of the disease. Given that the NCDB encompasses approximately 70% of newly diagnosed patients in the American population and comprises academic and non-academic institutions, high generalizability may be reasonably inferred [13].

## 5. Conclusions

In this large analysis, the use of perioperative chemotherapy was not associated with improved survival in rare subtypes of retroperitoneal sarcoma. However, selection bias must be considered when interpreting these findings.

## Figures and Tables

**Table 1:** Overall cohort demographic, clinical, and pathologic characteristics.

	Overall N = 851	No Chemotherapy N = 624	Chemotherapy N = 227	*p*-Value *
Age	62 (51, 71)	64 (55, 73)	56 (42, 64)	<0.001
Sex				0.9
Male	503 (59%)	368 (59%)	135 (59%)	
Female	348 (41%)	256 (41%)	92 (41%)	
Race				0.5
White	727 (85%)	535 (86%)	192 (85%)	
Black	75 (8.8%)	51 (8.2%)	24 (11%)	
Other	34 (4.0%)	25 (4.0%)	9 (4.0%)	
Histology				<0.001
Angiosarcoma	60 (7.1%)	33 (5.3%)	27 (12%)	
Undifferentiated pleomorphic sarcoma	303 (36%)	216 (35%)	87 (38%)	
Myxoid/round cell liposarcoma	246 (29%)	217 (35%)	29 (13%)	
Spindle cell sarcoma	192 (23%)	147 (24%)	45 (20%)	
Synovial sarcoma	50 (5.9%)	11 (1.8%)	39 (17%)	
Grade				<0.001
I	96 (15%)	90 (18%)	6 (4.1%)	
II	87 (14%)	70 (14%)	17 (11%)	
III	161 (25%)	115 (23%)	46 (31%)	
IV	156 (24%)	111 (23%)	45 (30%)	
Tumor size				0.006
<10 cm	205 (24%)	156 (25%)	49 (22%)	
10–20 cm	348 (41%)	240 (38%)	108 (48%)	
>20 cm	252 (30%)	200 (32%)	52 (23%)	
Radiotherapy	284 (33%)	210 (34%)	74 (33%)	0.8
Chemotherapy sequence				
Neoadjuvant chemotherapy	76 (8.9%)	-	76 (33%)	
Adjuvant chemotherapy	127 (14.9%)	-	127 (56%)	
Neoadjuvant and adjuvant chemotherapy	9 (1.5%)	-	9 (4.0%)	
Surgical margin				0.5
No residual tumor R0	400 (47%)	298 (48%)	102 (45%)	
Residual tumor R1or R2	129 (15%)	89 (14%)	40 (18%)	
Microscopic residual tumor R1	131 (15%)	92 (15%)	39 (17%)	
Macroscopic residual tumor R2	33 (3.9%)	24 (3.8%)	9 (4.0%)	

* *p*-values were derived using Student’s *t*-tests for continuous variables and chi-square tests for categorical variables.

**Table 2:** Multivariable-adjusted odds of treatment with chemotherapy in patients with retroperitoneal sarcoma.

	aHR * [95%-CI]	*p*-Value
Age (Reference: <60 years)		
Age (60–79 years)	0.56 [0.32–0.97]	0.04
Age (≥80 years)	0.18 [(0.04–0.82]	0.03
Sex (Reference: Female)		
Sex (Male)	0.96 [0.56–1.66]	0.9
Race (Reference: White)		
Race (Black)	1.42 [0.59–3.45]	0.44
Race (Other/Unknown)	2.66 [0.62–11.35]	0.19
Tumor Size (Reference: <10 cm)		
Tumor Size (10–20 cm)	1.09 [0.56–2.1]	0.80
Tumor Size (>20 cm)	0.82 [0.39–1.71]	0.59
Grade (Reference: Grade I/II)		
Grade (III/IV)	2.52 [1.35–4.69]	0.004
Margin (Reference: R0)		
Margin (R1)	1.50 [0.78–2.87]	0.22
Margin (R2)	1.17 [0.42–3.29]	0.76
Radiotherapy (Reference: No Radiotherapy)		
Radiotherapy (Yes)	1.22 [0.70–2.12]	0.48

* Multivariate logistic regression model adjusted for age, sex, race, tumor size, grade, margin status, and application of radiotherapy.

**Table 3:** Multivariable-adjusted Cox proportional hazards regression of death from retroperitoneal sarcoma in the overall cohort.

	aHR* [95%-CI]	*p*-Value
Chemotherapy (Reference: No Chemotherapy)		
Chemotherapy	0.89 [0.55–1.43]	0.63
Age (Reference: <60 years)		
Age (60–79 years)	1.55 [1.04–2.32]	0.03
Age (≥80 years)	5.05 [1.85–13.79]	0.002
Sex (Reference: Female)		
Sex (Male)	0.92 [0.63–1.34]	0.65
Tumor Size (Reference: <10 cm)		
Tumor size (10–20 cm)	0.77 [0.49–1.23]	0.28
Tumor size (>20 cm)	0.63 [0.36–1.10]	0.1
Grade (Reference: Grade I/II)		
Grade (III/IV)	0.93 [0.60–1.43]	0.73
Margin (Reference: R0)		
Margin (R1)	0.86 [0.50–1.50]	0.6
Margin (R2)	2.77 [1.18–6.53]	0.02
Radiotherapy (Reference: No Radiotherapy)		
Radiotherapy	1.20 [0.80–1.79]	0.38

* Multivariable Cox regression model adjusted for age, sex, tumor size, grade, margin status, and treatment with radiotherapy.

## Data Availability

Data available on request from the corresponding author.

## Supplementary Materials

The following supporting information can be downloaded at https://www.mdpi.com/article/10.3390/cancers17121931/s1. Figure S1: overall survival of patients with localized retroperitoneal sarcoma, stratified by receipt of neoadjuvant or adjuvant chemotherapy; Figure S2: jitter plots demonstrating sample balance before and after propensity score matching for age, sex, grade, margin status, and tumor size; Table S1: multivariable-adjusted Cox proportional hazards regression of death from retroperitoneal sarcoma in patients with undifferentiated pleomorphic sarcoma; and Table S2: multivariable-adjusted Cox proportional hazards regression of death from retroperitoneal sarcoma in patients with myxoid liposarcoma.

## References

[1] Leffall L.D. (1991). Retroperitoneal sarcoma. Ann. Surg..

[2] Schmitz E., Nessim C. (2022). Retroperitoneal Sarcoma Care in 2021. Cancers.

[3] Mendenhall W.M., Zlotecki R.A., Hochwald S.N., Hemming A.W., Grobmyer S.R., Cance W.G. (2005). Retroperitoneal soft tissue sarcoma. Cancer.

[4] Bonvalot S., Gronchi A., Le Péchoux C., Swallow C.J., Strauss D., Meeus P., van Coevorden F., Stoldt S., Stoeckle E., Rutkowski P. (2020). Preoperative radiotherapy plus surgery versus surgery alone for patients with primary retroperitoneal sarcoma (EORTC-62092: STRASS): A multicentre, open-label, randomised, phase 3 trial. Lancet Oncol..

[5] Almond L.M., Gronchi A., Strauss D., Jafri M., Ford S., Desai A. (2018). Neoadjuvant and adjuvant strategies in retroperitoneal sarcoma. Eur. J. Surg. Oncol..

[6] Datta J., Ecker B.L., Neuwirth M.G., Geha R.C., Fraker D.L., Roses R.E., Karakousis G.C. (2017). Contemporary reappraisal of the efficacy of adjuvant chemotherapy in resected retroperitoneal sarcoma: Evidence from a nationwide clinical oncology database and review of the literature. Surg. Oncol..

[7] Li X., Wu T., Xiao M., Wu S., Min L., Luo C. (2021). Adjuvant therapy for retroperitoneal sarcoma: A meta-analysis. Radiat. Oncol..

[8] Miura J.T., Charlson J., Gamblin T.C., Eastwood D., Banerjee A., Johnston F.M., Turaga K.K. (2015). Impact of chemotherapy on survival in surgically resected retroperitoneal sarcoma. Eur. J. Surg. Oncol..

[9] Angele M.K., Albertsmeier M., Prix N.J., Hohenberger P., Abdel-Rahman S., Dieterle N., Schmidt M., Mansmann U., Bruns C.J., Issels R.D. (2014). Effectiveness of regional hyperthermia with chemotherapy for high-risk retroperitoneal and abdominal soft-tissue sarcoma after complete surgical resection: A subgroup analysis of a randomized phase-III multicenter study. Ann. Surg..

[10] Zhou D.D., Connolly E.A., Mar J., Lazarakis S., Grimison P.S., Connor J., Gyorki D.E., Hong A.M. (2024). A systematic review of the role of chemotherapy in retroperitoneal sarcoma by the Australia and New Zealand sarcoma association clinical practice guidelines working party. Cancer Treat. Rev..

[11] Gronchi A., Miah A.B., Dei Tos A.P., Abecassis N., Bajpai J., Bauer S., Biagini R., Bielack S., Blay J.Y., Bolle S. (2021). Soft tissue and visceral sarcomas: ESMO-EURACAN-GENTURIS Clinical Practice Guidelines for diagnosis, treatment and follow-up. Ann. Oncol..

[12] Lambdin J., Ryan C., Gregory S., Cardona K., Hernandez J.M., van Houdt W.J., Gronchi A. (2023). A Randomized Phase III Study of Neoadjuvant Chemotherapy Followed by Surgery Versus Surgery Alone for Patients with High-Risk Retroperitoneal Sarcoma (STRASS2). Ann. Surg. Oncol..

[13] Bilimoria K.Y., Stewart A.K., Winchester D.P., Ko C.Y. (2008). The National Cancer Data Base: A powerful initiative to improve cancer care in the United States. Ann. Surg. Oncol..

[14] Rosenbaum P., Rubin D. (1983). The Central Role of the Propensity Score in Observational Studies For Causal Effects. Biometrika.

[15] Tortorello G.N., Li E.H., Sharon C.E., Ma K.L., Maki R.G., Miura J.T., Fraker D.L., DeMatteo R.P., Karakousis G.C. (2023). Neoadjuvant Chemotherapy in Retroperitoneal Sarcoma: A National Cohort Study. Ann. Surg. Oncol..

[16] von Mehren M., Kane J.M., Agulnik M., Bui M.M., Carr-Ascher J., Choy E., Connelly M., Dry S., Ganjoo K.N., Gonzalez R.J. (2022). Soft Tissue Sarcoma, Version 2.2022, NCCN Clinical Practice Guidelines in Oncology. J. Natl. Compr. Cancer Netw..

[17] Gortzak E., Azzarelli A., Buesa J., Bramwell V.H., van Coevorden F., van Geel A.N., Ezzat A., Santoro A., Oosterhuis J.W., van Glabbeke M. (2001). A randomised phase II study on neo-adjuvant chemotherapy for ‘high-risk’ adult soft-tissue sarcoma. Eur. J. Cancer.

[18] Frustaci S., Gherlinzoni F., De Paoli A., Bonetti M., Azzarelli A., Comandone A., Olmi P., Buonadonna A., Pignatti G., Barbieri E. (2001). Adjuvant chemotherapy for adult soft tissue sarcomas of the extremities and girdles: Results of the Italian randomized cooperative trial. J. Clin. Oncol..

[19] Vlenterie M., Litière S., Rizzo E., Marréaud S., Judson I., Gelderblom H., Le Cesne A., Wardelmann E., Messiou C., Gronchi A. (2016). Outcome of chemotherapy in advanced synovial sarcoma patients: Review of 15 clinical trials from the European Organisation for Research and Treatment of Cancer Soft Tissue and Bone Sarcoma Group; setting a new landmark for studies in this entity. Eur. J. Cancer.

[20] Gazendam A.M., Popovic S., Munir S., Parasu N., Wilson D., Ghert M. (2021). Synovial Sarcoma: A Clinical Review. Curr. Oncol..

[21] Eilber F.C., Brennan M.F., Eilber F.R., Eckardt J.J., Grobmyer S.R., Riedel E., Forscher C., Maki R.G., Singer S. (2007). Chemotherapy is associated with improved survival in adult patients with primary extremity synovial sarcoma. Ann. Surg..

[22] Gamboa A.C., Gronchi A., Cardona K. (2020). Soft-tissue sarcoma in adults: An update on the current state of histiotype-specific management in an era of personalized medicine. CA Cancer J. Clin..

[23] Avancès C., Mottet N., Mahatmat A., Chapuis E., Serre I., Culine S. (2006). Prognostic factors for first recurrence in patients with retroperitoneal sarcoma. Urol. Oncol..

[24] Anaya D.A., Lahat G., Wang X., Xiao L., Pisters P.W., Cormier J.N., Hunt K.K., Feig B.W., Lev D.C., Pollock R.E. (2010). Postoperative nomogram for survival of patients with retroperitoneal sarcoma treated with curative intent. Ann. Oncol..

[25] Ardoino I., Miceli R., Berselli M., Mariani L., Biganzoli E., Fiore M., Collini P., Stacchiotti S., Casali P.G., Gronchi A. (2010). Histology-specific nomogram for primary retroperitoneal soft tissue sarcoma. Cancer.

[26] Gronchi A., Miceli R., Shurell E., Eilber F.C., Eilber F.R., Anaya D.A., Kattan M.W., Honoré C., Lev D.C., Colombo C. (2013). Outcome prediction in primary resected retroperitoneal soft tissue sarcoma: Histology-specific overall survival and disease-free survival nomograms built on major sarcoma center data sets. J. Clin. Oncol..

[27] Borghi A., Gronchi A. (2024). Sarculator: How to improve further prognostication of all sarcomas. Curr. Opin. Oncol..

[28] Squires M.H., Ethun C.G., Donahue E.E., Benbow J.H., Anderson C.J., Jagosky M.H., Manandhar M., Patt J.C., Kneisl J.S., Salo J.C. (2022). Extremity Soft Tissue Sarcoma: A Multi-Institutional Validation of Prognostic Nomograms. Ann. Surg. Oncol..

[29] de Bree E., Michelakis D., Heretis I., Kontopodis N., Spanakis K., Lagoudaki E., Tolia M., Zografakis-Sfakianakis M., Ioannou C., Mavroudis D. (2023). Retroperitoneal Soft Tissue Sarcoma: Emerging Therapeutic Strategies. Cancers.

[30] Gronchi A., Ferrari S., Quagliuolo V., Broto J.M., Pousa A.L., Grignani G., Basso U., Blay J.-Y., Tendero O., Beveridge R.D. (2017). Histotype-tailored neoadjuvant chemotherapy versus standard chemotherapy in patients with high-risk soft-tissue sarcomas (ISG-STS 1001): An international, open-label, randomised, controlled, phase 3, multicentre trial. Lancet Oncol..

