# Supplementary material for: Perioperative Systemic Therapy in Rare, Chemosensitive Subtypes of Retroperitoneal Sarcoma: A Hospital-Based Propensity Score-Matched Analysis
Source: Cancers (Basel). 2025 Jun 10;17(12):1931. doi: 10.3390/cancers17121931 (PMC12190581; doi:10.3390/cancers17121931)

SUPPLEMENTARY MATERIAL

Figure S1: Overall survival of patients with localised retroperitoneal sarcoma, stratified by receipt of neoadjuvant or adjuvant chemotherapy.

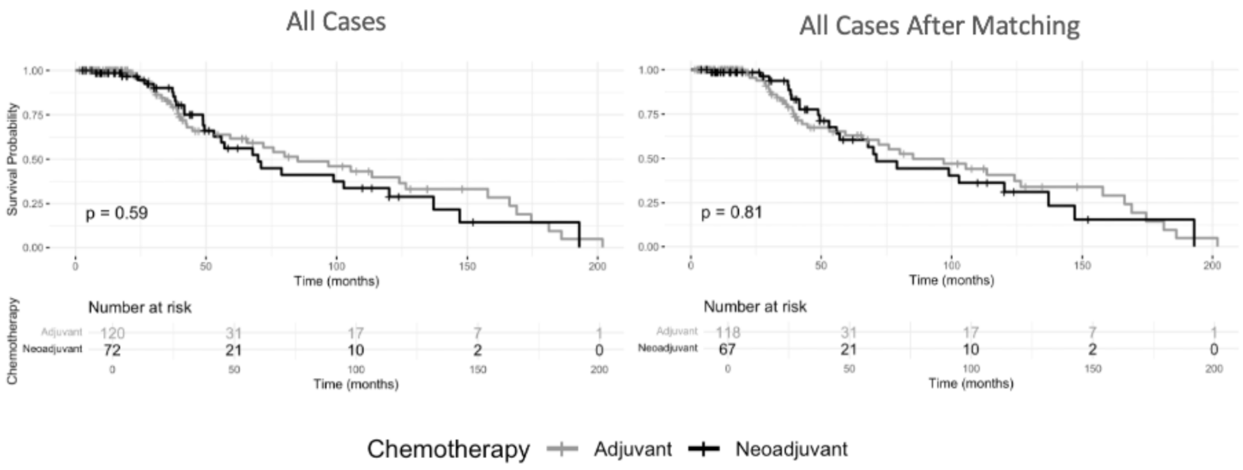

Supplement: Supplementary file 1 [file cancers-17-01931-s001.zip › Figure_S1.pdf]
