# Supplementary material for: Perioperative Systemic Therapy in Rare, Chemosensitive Subtypes of Retroperitoneal Sarcoma: A Hospital-Based Propensity Score-Matched Analysis
Source: Cancers (Basel). 2025 Jun 10;17(12):1931. doi: 10.3390/cancers17121931 (PMC12190581; doi:10.3390/cancers17121931)
Supplement: Supplementary file 1 [file cancers-17-01931-s001.zip › Table_S1.pdf]

SUPPLEMENTARY MATERIAL

Table S1: Multivariable-adjusted Cox proportional hazards regression of death from retroperitoneal sarcoma in patients with undifferentiated pleomorphic sarcoma

Variable	aHR* [95%-CI]	p-value
Chemotherapy (Reference: No Chemotherapy)		
Chemotherapy	1.43 [0.58 - 3.50]	0.44
Age (Reference: <60 years)		
Age (60-79 years)	1.88 [0.84 - 4.20]	0.12
Age (≥80 years)	5.73 [0.45 - 72.94]	0.18
Sex (Reference: Female)		
Sex (Male)	1.01 [0.46 - 2.23]	0.97
Tumor size (Reference: <10 cm)		
Tumor size (10-20 cm)	0.69 [0.29 - 1.66]	0.41
Tumor size (>20 cm)	0.87 [0.27 - 2.78]	0.81
Grade (Reference: I/II)		
Grade (III/IV)	1.33 [0.23 - 7.71]	0.75
Margin (Reference: R0)		
Margin (R1)	4.17 [1.14 - 15.25]	0.03
Margin (R2)	3.97 [1.18 - 13.42]	0.03
Radiotherapy (Reference: No Radiotherapy)		
Radiotherapy	1.21 [0.51 - 2.87]	0.66

* Multivariate COX regression model adjusted for: Age, Sex, Tumor size, Grade, Margin status, and Application of radiotherapy.
