# Supplementary material for: Perioperative Systemic Therapy in Rare, Chemosensitive Subtypes of Retroperitoneal Sarcoma: A Hospital-Based Propensity Score-Matched Analysis
Source: Cancers (Basel). 2025 Jun 10;17(12):1931. doi: 10.3390/cancers17121931 (PMC12190581; doi:10.3390/cancers17121931)
Supplement: Supplementary file 1 [file cancers-17-01931-s001.zip › Table_S2.pdf]

SUPPLEMENTARY MATERIAL

Table S2: Multivariable-adjusted Cox proportional hazards regression of death from retroperitoneal sarcoma in patients with myxoid liposarcoma

Variable	aHR* [95%-CI]	p-value
Chemotherapy (Reference: No Chemotherapy)		
Chemotherapy	1.04 [0.43 - 2.49]	0.93
Age (Reference: <60 years)		
Age (60-79 years)	0.76 [0.37 - 1.54]	0.44
Age (≥80 years)	3.38 [0.77 - 14.78]	0.11
Sex (Reference: Female)		
Sex (Male)	0.96 [0.50 - 1.86]	0.91
Tumor size (Reference <10 cm)		
Tumor size (10-20 cm)	0.73 [0.23 - 2.34]	0.59
Tumor size (>20 cm)	0.86 [0.30 - 2.43]	0.77
Grade (Reference: I/II)		
Grade (III/IV)	1.67 [0.67 - 4.13]	0.30
Margin (Reference: R0)		
Margin (R1)	0.66 [0.30 - 1.49]	0.32
Margin (R2)	4.30 [0.86 - 21.62]	0.08
Radiotherapy (Reference: No Radiotherapy)		
Radiotherapy	0.97 [0.46 - 2.04]	0.93

* Multivariate COX regression model adjusted for: Age, Sex, Tumor size, Grade, Margin status, and Application of radiotherapy.
